# A cross dataset meta-model for hepatitis C detection using multi-dimensional pre-clustering

**DOI:** 10.1038/s41598-025-91298-0

**Published:** 2025-03-01

**Authors:** Aryan Sharma, Tanmay Khade, Shashank Mouli Satapathy

**Affiliations:** School of Computer Science and Engineering, Vellore Institute of Technology, Vellore, Tamil Nadu 632014 India

**Keywords:** Hepatitis C, Clustering, K-centroid clustering, K-means clustering, K-modes clustering, Machine learning, Stacking meta-model, XGBoost, KNN, SVM, RF, Diseases, Medical research

## Abstract

Hepatitis C is a liver infection triggered by the hepatitis C virus (HCV). The infection results in swelling and irritation of the liver, which is called inflammation. Prolonged untreated exposure to the virus can lead to chronic hepatitis C. This can result in serious health complications such as liver damage, hepatocellular carcinoma (HCC), and potentially death. Therefore, rapid diagnosis and prompt treatment of HCV is crucial. This study utilizes machine learning (ML) to precisely identify hepatitis C in patients by analyzing parameters obtained from a standard biochemistry test. A hybrid dataset was acquired by merging two commonly used datasets from individual sources. A portion of the dataset was used as a hold-out set to simulate real-world data. A multi-dimensional pre-clustering approach was used in this study in the form of k-means for binning and k-modes for categorical clustering. The pre-clustering approach was used to extract a new feature. This extracted feature column was added to the original dataset and was used to train a stacked meta-model. The model was compared against baseline models. The predictions were further elaborated using explainable artificial intelligence. The models used were XGBoost, K-nearest neighbor, support vector classifier, and random forest (RF). The baseline score obtained was 94.25% using RF, while the meta-model gave a score of 94.82%.

## Introduction

Hepatitis remains a significant global healthcare challenge, with an estimated 354 million people affected worldwide. It exhibits a mortality rate surpassing that of any other chronic disease. The hepatitis family comprises five strains of the virus; these are Hepatitis A, B, C, D, and E. While all the strains of hepatitis contribute to liver damage, they differ in their modes of transmission and prevention methods^1^. In 2023, a WHO survey estimated a global count of 58 million people who chronically suffer from Hepatitis C with about 3.2 million of these being adolescents and children, this number rises every year by about 1.5 million^2^. Cirrhosis and hepatocellular carcinoma caused an estimated 290,000 deaths in 2019. Fortunately, 95% of these cases can be effectively cured within the first 8–12 weeks of diagnosis through direct-acting antiviral medicines (DAAs)^3^. Liver damage can be avoided by rapid diagnosis of the virus. The primary stage of Hepatitis C is known as acid hepatitis and can last for around five months with minimal symptoms. Beyond this period, the disease becomes critical and leads to long-term sickness. If left untreated in the early stages, the latter stages may lead to liver scarring, fibrosis, and cirrhosis, which may cause liver failure and HCC. Currently, vaccines are not available to prevent the spread of hepatitis c, unlike the other variants of viral hepatitis^4^.

Due to the mild nature of the symptoms, patients often only recognize them when the infection has become chronic. Hepatitis C symptoms include abdominal pain, jaundice (yellowing of the skin and eyes), weight loss, dark-coloured urine, and fatigue. Bloodborne pathogens cause HCV. Common ways of contracting the virus include sharing toothbrushes, razors, scissors, unhygienic tattooing or piercing equipment, and unscreened blood transfusions. Sharing needles and other equipment for preparing and injecting drugs is the primary source of transmission of the virus^3^. The causes of Hep C can be studied further using the Mendelian randomization technique^5^. The challenge of predicting treatment outcomes benefits greatly from advances in machine learning, particularly in complex biomedical contexts such as drug-food interactions and disease-associated variant localization^6,7^. According to a 2017 WHO study, only 20% of HCV-infected patients were diagnosed, and only 15% of those diagnosed received treatment. Thus, HCV infection continues to be a leading cause of liver disease worldwide^2^.

Currently, there are two primary methods for diagnosing HCV. A serological test is used to detect the presence of the disease, while a virological test is necessary to confirm and monitor the infection. The HCV antibody test, commonly called the anti-HCV test, is a blood test to determine if a person has ever contracted HCV. This test checks for antibodies in the bloodstream, indicating previous exposure to the virus. The presence of antibodies confirms a past infection but is not feasible to ascertain its current presence. It is possible that an infection had developed in the past but was cured, resulting in the formation of these antibodies. An HCV RNA PCR test is thus required to confirm the presence of the virus in the bloodstream^8^.

In recent years, machine learning techniques have revolutionized the landscape of predictive analytics in healthcare, offering opportunities to understand and implement these techniques for the welfare of patients. Selecting the correct parameters and proper data analysis are essential aspects of an accurate prediction. Past studies have proven that using static patient records along with ML models can help accurately predict the presence of the disease. Existing research has employed supervised models such as K-nearest neighbor (KNN), support vector machine (SVM), Naïve Bayes, random forests, decision trees, etc. Machine learning combines techniques from computer science and mathematics to devise algorithms. These algorithms take in static data from laboratories and learn patterns and relationships between them. This learned information is then used to predict the required ground truth label. The workings of the models can be further explored using XAI tools. XAI tools are used to understand the individual, feature-wise contribution to the model’s prediction.

This research proposes using a multi-dimensional pre-clustering approach followed by supervised ML to improve the convergence of the results. Throughout this study, we aim to avoid over-optimistic results by preventing data leakage. The study explores applying unsupervised approaches to generate new features from the dataset. Our study aims to achieve the following:

- Utilize a large, diverse, hybrid dataset by merging two widely used datasets.
- Evaluate the efficacy of unsupervised pre-clustering techniques against traditional ML methods.
- Develop a stacked meta-model to create an ensemble of various ML algorithms.
- Employ Explainable AI techniques to understand the contribution of each parameter in the final prediction.
- Conduct two experiments to evaluate the suggested model’s effectiveness. We would utilize baseline ML models without using pre-clustering in the first experiment. In the next experiment, pre-clustering would be followed by the same ML models. Further, a stacked meta-model would be constructed and compared to the previous models.

## Literature survey

A systemic literature review of research papers was conducted to explore recent advancements in machine learning (ML) methodologies for predicting HCV infection. As elaborated in the following sections, several noteworthy research papers employed multiple novel techniques. Common concepts across multiple research papers were also identified and discussed below. This literature review helped us identify the existing gaps in the current studies and formulate an approach to address them.

### Noteable research papers

A study by Farghaly et al.^9^ investigates feature selection results on the model’s overall accuracy. The study utilizes the Naïve–Bayes, random forests, KNN, and logistic regression models. The study compares the efficacy of sequential forward selection (SFS) for feature selection against no feature selection. The result confirms a more accurate result can be obtained by using fewer features and conducting hyperparameter tuning. Their data included 859 patient data points with 12 features.

Alizargar et al.^10^ conducted a comparative study on two open-source datasets from the University of California, Irvine (UCI) and the National Health and Nutrition Examination Survey (NHANES). The study used preprocessing techniques such as median imputation on missing values. This study explained the importance of using median imputation to gain more accurate and reliable results. The study additionally applies an embedded method for feature selection to pick out the most pertinent features during model training. This algorithm is directly incorporated as a part of model training rather than a part of data preprocessing. The study used six ML models: artificial neural networks (ANN), SVM, XGboost, Logistic regression, KNN, and decision trees. A maximum accuracy of 95% was achieved using both XGboost and SVM models.

Alotaibi et al.^11^ conducted a study that used the UCI dataset, which contains data on patients in Egypt. The study used preprocessing techniques like the interquartile range for outlier detection and removal, random oversampling to balance the dataset, and SFS for feature selection. The study used a tenfold cross-validation, sampled using a stratified method using GridSearchCV to train the models with hyperparameter tuning. Random forest, XGBoost, and extra tree models were used for training and evaluation. The research also integrated eXplainable AI (XAI) methods such as SHapley Additive exPlanations (SHAP) and Local Interpretable Model-agnostic Explanations (LIME) to foster trust among medical practitioners. The extra decision tree model achieved the highest accuracy in the study, at 96.92%.

Lilhore et al.^12^ conducted a study that explored a hybrid predictive model and used the rankers method for feature selection. Their proposed model employed the ranker method for selecting features and merges an iterative strategy with Random Forest and Support Vector Machine models. SMOTE was applied to the data and the maximum accuracy achieved was 96.82%.

Naeem et al.^13^ studied the spread of HCV in pregnant women. The study confirmed that pregnant women residing in rural areas are more prone to HCV than those living in urban regions. A questionnaire was used to acquire data from the patients. The study uses SVM, Naive Bayes, and KNN ML models to predict HCV among pregnant women using the WEKA tool. The study achieves an accuracy of 86.32% using the SVM model.

### Additional findings

Various studies utilized ANN to predict HCV. ANN is a deep learning model that tries to imitate a human brain. Zabara et al.^14^ achieved 100% accuracy on a dataset of 90 hepatitis C patients from Romania using the ANN model. Butt et al.^15^ and Syafaâ et al.^16^ achieved 98.89% precision and 95.12% accuracy, respectively, by implementing the ANN model on a dataset from the UCI ML repository. Kaunang et al.^17^ implemented logistic regression on the same data to acquire an accuracy of 97.9%. KNN is another prominent model that is frequently used to predict HCV. Harabor et al.^18^ acquired an accuracy of 98.1% by implementing KNN on a dataset from Romania. A similar study by Nandipati et al.^19^ on a dataset from UCI for Egyptian patients implemented KNN to achieve a 51.06% accuracy. Dagan et al.^20^ utilized XGBoost on Israel’s largest healthcare organization’s dataset to acquire 0.95 area under the ROC curve. Kashif et al.^21^ applied a decision tree model to achieve 85.9155% accuracy on data from Lahore’s Hospital. McCandlish et al.^22^ investigated the application of the ANN model on a synthetically generated dataset to achieve 99% $R^2$ values. SMOTE is a data preprocessing concept implemented in many recent studies^12,23–27^. SMOTE is applied to a dataset that has a minority class with fewer data points than the majority class. In the study concerning HCV prediction, the patients diagnosed with HCV infection were identified as the minority class. SMOTE provides solutions for data imbalance and visual representation. SMOTE improves the model’s accuracy as additional data points from minority classes are created for training. Tao wang et al.^28^ use a Louvain community detection algorithm for finding the optimal number of clusters. An efficient deep learning based method has been used to handle missing value imputations^29^.

### Explainable artificial intelligence

XAI approach is applied to elaborate on the results generated by the machine learning models. The ever-increasing complexity of machine learning models creates a gap in human understanding of the model. XAI helps tackle this challenge by presenting a human-understandable explanation for the results generated by the model. Two widely used libraries implement the XAI approach: SHAP and LIME. XAI techniques come in two types: model-agnostic and model-specific. The model-agnostic method can be applied to any machine learning model, irrespective of its structure or type.

Meanwhile, model-specific methods can be implemented only for specific categories of models. DeepLIFT and Grad-CAM are the libraries that can be leveraged to implement XAI on deep learning models. Both SHAP and LIME are model-agnostic methods of XAI. SHapley’s Additive explanation explains the prediction by computing the contribution of each feature. Local Interpretable Model-agnostic Explanations focus on explaining the model’s prediction for a specific instance^11,24,30^.

### Research gaps

After conducting a thorough literature review, the following research gaps were analyzed:

- Overlooks the opportunity to combine the UCI and NHANES datasets to form a more realistic hybrid dataset^23^. It becomes challenging for professionals to generalize the results of just one dataset.
- The conversion of continuous data into categorical data using data binning for applying an unsupervised machine learning approach was not explored in any research.
- The test-train split stage was not clearly articulated. The stage of the test-train split determines the risk of data leakage in the training data. This misses creating real-world unseen data for testing of the model.

### Our contributions

- Our process uses a collaboration of supervised and unsupervised machine learning techniques to detect hepatitis C accurately. Unsupervised learning helps aggregate large amounts of data into more understandable clusters.
- We have combined the two existing open-source datasets to create a robust and more reliable hybrid dataset. The datasets were merged using the common parameters. The datasets were extracted from the University of California, Irvine, and NHANES.
- Use of Stratified tenfold GridSearchCV for hyperparameter tuning. The four algorithms trained and compared for the above method are XGBoost, KNN, RF, and SVMs.
- We convert the continuous data into categorical data by using k-means to split the data into clusters and, by extension, group the data together based on the similarity of the data.
- Categorical data is acquired after binning the data. Then, k-mode clustering is utilized with Cao initialization to cluster this data further. We use the elbow method and silhouette score to find the optimal number and location of the bins in k-means. The elbow, silhouette scores, and the Davies-Bouldin methods identify the ideal number of clusters.
- Utilized a meta-model by stacking various ML techniques to overcome their shortcomings.
- Explained the predictions of our model through feature contribution using SHAP.

## Material and methods

### Datasets

A hybrid dataset was curated for this study. The UCI dataset consisted of data from 615 patients with thirteen parameters. Lichtinghagen et al.^31^ created and donated the dataset to the Machine Learning Repository of the University of California, Irvine (UCI). This data included laboratory values as well as demographic data. The thirteen parameters were Age, Gender, Albumin(ALB), Alkaline phosphatase (ALP), Aspartate transaminase (AST), Bilirubin (BIL), Cholinesterase (CHE), Cholesterol (CHOL), Creatinine (CREA), Gamma-glutamyltransferase (GGT), Total Protein (PROT), and the ground truth feature categorizes patients as either positive or negative for hepatitis C. All values except the target variable were numerical. The features and descriptions are shown in Table 1.

**Table 1:** Feature description for UCI dataset.

Feature	Count	Average ($\overline{x}$)	$\sigma$	Min. value	$Q_1$	$Q_2$	$Q_3$	Max. value
HepC	615.00	1.87	0.32	1.00	2.00	2.00	2.00	2.00
Age (in months)	615.00	568.89	120.66	228.00	468.00	564.00	648.00	924.00
Gender	615.00	1.38	0.48	1.00	1.00	1.00	2.00	2.00
ALB (g/L)	614.00	41.62	5.78	14.90	38.80	41.95	45.20	82.20
ALP (IU/L)	597.00	68.28	26.02	11.30	52.50	66.20	80.10	416.60
ALT (U/L)	614.00	28.45	25.46	0.90	16.40	23.00	33.07	325.30
AST (U/L)	615.00	34.78	33.09	10.60	21.60	25.90	32.90	324.00
BIL(umol/L)	615.00	11.39	19.67	0.80	5.30	7.30	11.20	254.00
CHE	615.00	8.19	2.20	1.42	6.93	8.26	9.59	16.41
CHOL (mmol/L)	605.00	5.36	1.13	1.43	4.61	5.30	6.06	9.67
CREA (umol/L)	615.00	81.28	49.75	8.00	67.00	77.00	88.00	1079.10
GGT (IU/L)	615.00	39.53	54.66	4.50	15.70	23.30	40.20	650.90
PROT (g/L)	614.00	72.04	5.40	44.80	69.30	72.20	75.40	90.00

The NHANES dataset^32,33^ consisted of data from 254 patients with eleven parameters obtained from a standard biochemistry blood test merged with demographic data. The data was sourced from the National Health and Nutrition Examination Survey (NHANES) conducted by the Centers for Disease Control and Prevention (CDC) . The eleven parameters utilized were Age, Gender, ALB (g/L), ALP (IU/L), ALT (U/L), AST (U/L), CHOL (mmol/L), CREA (umol/L), GGT (IU/L), PROT (g/L) and the target variable which categorized the patient based on the presence or absence of hepatitis C, tested through an HCV-RNA test. All values except gender and the target variable were numeric. The features and their descriptions are shown in Table 2.

The two datasets were hybridized into one dataset with 869 patient entries and thirteen parameters. Table 3 displays the dataset description and class details. Figure 3 shows the class-wise distribution of the data. The given parameter and its count are displayed by the x-axis and y-axis, respectively. The hybrid dataset’s features and descriptions are shown in Table 3.

**Table 2:** Feature description for NHANES dataset.

Feature	Count	Average ($\overline{x}$)	$\sigma$	Min. value	$Q_1$	$Q_2$	$Q_3$	Max. value
HepC	254.00	1.64	0.47	1.00	1.00	2.00	2.00	2.00
Age (in months)	254.00	677.66	187.26	144.00	603.00	726.00	789.00	960.00
Gender	254.00	1.38	0.48	1.00	1.00	1.00	2.00	2.00
ALB (g/L)	250.00	39.37	4.05	26.00	37.00	40.00	42.00	49.00
ALP (IU/L)	250.00	87.31	37.99	36.00	66.00	80.00	97.00	363.00
ALT (U/L)	250.00	32.44	36.50	3.00	13.00	21.00	37.00	338.00
AST (U/L)	249.00	33.28	31.78	6.00	18.00	23.00	36.00	307.00
CHOL (umol/L)	250.00	4.52	1.09	2.09	3.75	4.44	5.17	8.22
CREA (umol/L)	250.00	95.26	92.34	38.01	66.30	79.56	93.70	968.86
GGT (IU/L)	250.00	55.77	77.21	5.00	17.00	28.00	52.00	550.00
PROT (g/L)	250.00	73.42	5.40	60.00	70.00	73.00	77.00	88.00

A multi-source dataset has been used to produce a more generalizable result. This hybrid dataset is believed to be a balanced representative of hepatitis C patient data and can help mitigate biases by overfitting a particular class.

It could be argued that due to the lack of a perfect overlap between the features of the two datasets, hybridizing them could result in several missing values that may hamper the training of ML models. Still, appropriate measures have been implemented to ensure that does not happen. These measures are included in the preprocessing section of this study and include imputation of the missing values through medians. Outlier treatment has also been performed to address this issue. These measures and their entire methodology are explained in further detail in the forthcoming section.

It could be argued that removing the total bilirubin parameter from the NHANES dataset could be detrimental to the predictions made by the model. However, a Han Ah Lee et al. study demonstrated that direct bilirubin was more predictive of prognosis in patients with cirrhosis than total bilirubin^34.^

**Table 3:** Feature description for the hybrid dataset.

Feature	Count	Average ($\overline{x}$)	$\sigma$	Min. value	$Q_1$	$Q_2$	$Q_3$	Max. value
HepC	869.00	1.81	0.39	1.00	2.00	2.00	2.00	2.00
Age (in years)	869.00	50.05	12.62	12.00	41.00	50.00	59.00	80.00
Gender	869.00	1.38	0.48	1.00	1.00	1.00	2.00	2.00
ALB (g/L)	864.00	40.96	5.43	14.90	38.80	41.00	44.40	82.20
ALP (IU/L)	847.00	73.90	31.26	11.30	56.40	70.00	84.30	416.60
ALT (U/L)	864.00	29.60	29.12	0.90	15.40	22.70	34.00	338.00
AST (U/L)	864.00	34.35	32.70	6.00	20.00	25.30	33.15	324.00
BIL(umol/L)	615.00	11.39	19.67	0.80	5.30	7.30	11.20	254.00
CHE	615.00	8.19	2.20	1.42	6.93	8.26	9.59	16.41
CHOL (mmol/L)	855.00	5.12	1.18	1.43	4.33	5.05	5.90	9.67
CREA (umol/L)	865.00	85.32	65.24	8.00	67.00	77.00	89.28	1079.10
GGT (IU/L)	865.00	44.22	62.41	4.50	16.00	24.20	43.90	650.90
PROT (g/L)	864.00	72.44	5.43	44.80	69.40	72.30	75.80	90.00

### Research questions

This study aims to answer the following questions during its completion:

- Would using pre-clustering provide a higher accuracy than a purely supervised machine learning approach?
- Would it be possible to demystify the black-box operations of the proposed method?
- Would a hybrid meta-model perform better than applying individual models?
- Could our proposed approach provide a more generalizable model?

### Experiments

**Fig. 1:**
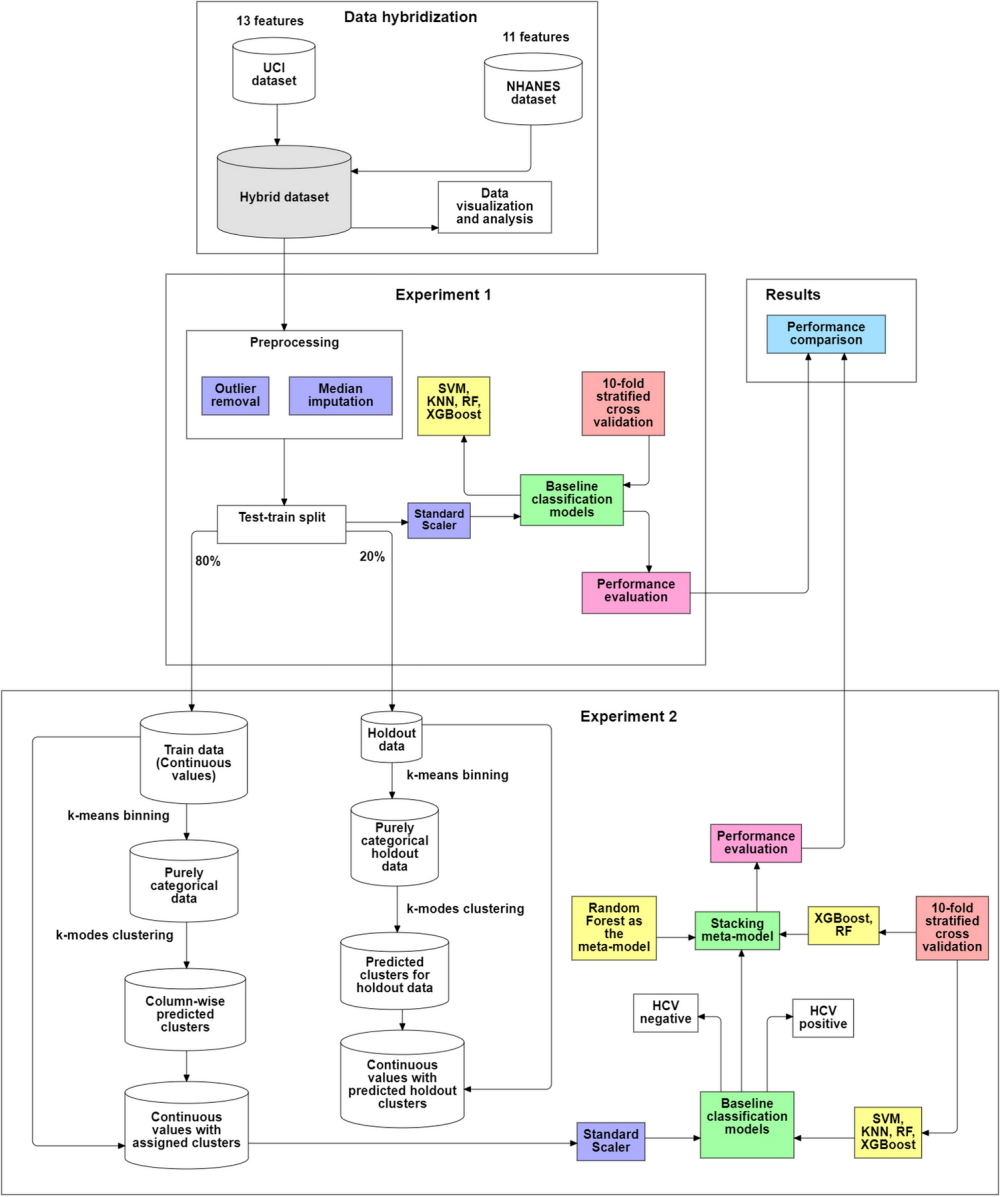
Diagrammatic representation of the process followed by this study.

In this section, we performed two experiments, the first experiment aimed to set a baseline performance for the ML models. The models were trained on pre-processed data and their performance was evaluated. In the second experiment, we aimed to utilize the proposed clustering approach for feature extraction. The introduction of these features improved the performance of the first experiment. The second experiment was vital in identifying how the proposed approach affected the outcome. Additionally, it studied the ability of a stacked meta-model to improve the accuracy of the achieved results. Throughout the experiments, each step is carefully analyzed to avoid data leakage. Finally, an ablation study was conducted to demonstrate the effectiveness of individual models. These experiments are expanded in further detail in the following sections. Figure 1 provides an overview of the entire process.

#### Experiment 1: Baseline evaluation

This experiment was subdivided into two phases. The pre-processing phase included data clean-up and the model implementation phase, during which various ML models were implemented. In the first phase, we addressed the problem of missing values and outliers. The two datasets used in this study differed in the number of features. Additionally, the features in the hybrid dataset had outliers, which were treated before applying machine learning techniques. We split the data post-clean-up and kept a ’holdout set’ aside for future evaluation. This holdout set is used for performance evaluation in the second phase. The following sections “Missing value imputation”, “Outlier detection and treatment”, and “Implementing ML models” explain these phases in detail.

The scores obtained from this experiment are considered baseline scores throughout this study. In the upcoming sections, these baseline scores are compared with the scores obtained from the proposed model.

##### Missing value imputation

Imputation refers to filling in missing values in a dataset using statistical estimates, usually of central tendencies. This study utilized the median as the measure of central tendency for imputation. Every occurrence of a missing (NA) value is replaced with the median of the observations. The distribution of features in our dataset is observed to be skewed. Additionally, it’s noted that the missing values appear to be random, indicating that they are not directly linked to specific patients or patient groups. Moreover, our dataset contains a considerable number of outliers. Considering these reasons, this research deemed the usage of median imputation appropriate. This method also gave us the best performance during mode evaluation. As a means of additional justification, many medical studies have been observed to employ median imputation to deal with missing values.

##### Outlier detection and treatment

An outlier is an observation or subset of observations that seem to diverge from the remaining data points significantly. The inconsistencies might arise from incorrect records, measurement errors, rare events, or biological extremities. Since ML relies on pattern learning, the existence of outliers can significantly deteriorate the model’s accuracy. This study uses an interquartile range (IQR) approach to deal with outliers. The outliers from all the variables outside the 2.5% to 97.5% range have been replaced with the lower and upper limits, respectively. The lower and upper limits are mathematically defined as shown in Eqs. (1) and (2)

1 $$upper = q3 + (1.5 \times iqr)$$

2 $$lower = q1 - (1.5 \times iqr)$$

where q1 and q3 represent the first and third quartiles, IQR is their difference.

The sensitivity of the limits was chosen as 1.5 after analysis of the data. An IQR approach is less sensitive to extreme values than its alternatives as it focuses only on the middle 50% of the data distribution. Additionally, our data is skewed and observed to be non-normal. An IQR method shows remarkable resilience against data skewness while maintaining the original integrity of the dataset. Additionally, the IQR method had the highest performance among the other techniques during testing^35^. We detected an aggregate of 6.004% outliers in the dataset. The highest number of outliers was found in the attribute bilirubin, with 98 outliers (11.277%), and the lowest was Age, with four outliers (0.46%). Table 4 shows the total number of outliers, the total number of values before, and the total number of values after the treatment process. Since none of the outliers have been removed, we see no change in the number of observations in rows 2 and 3. The importance of this outlier imputation process is further explored in “Preprocessing impact study”.

**Table 4:** Tabular overview of outlier imputation.

	Age	ALB	ALP	ALT	AST	BIL	CHE	CHOL	CREA	GGT	PROT	Total
Outlier count	4	28	34	64	93	98	81	12	38	97	25	574
Total before imputation	869	869	869	869	869	869	869	869	869	869	869	9559
Total after imputation	869	869	869	869	869	869	869	869	869	869	869	9559

##### Feature scaling

We used a normalization process called standard scaling to scale the features before passing them through the models. Standard scaling nullifies the mean and scales the data to have a unit variance. The majority of the algorithms benefit from this process as it reduces the number of large-value calculations, speeding up the convergence and increasing the chance for higher accuracy. All features with an integer value have gone through this scaling process. These features were: Age (in years), Gender, ALB (g/L), ALP (IU/L), ALT (U/L), AST (U/L), BIL(umol/L), CHE, CHOL (mmol/L), CREA (umol/L), GGT (IU/L), PROT (g/L), “kmodes_predicted”. This process was used in all experiments in this manuscript. We further explored the efficacy of this step in the section “Preprocessing impact study”.

##### Implementing ML models

The data obtained in the previous steps was used to train the following standard ML models: XGBoost, KNN, RF, and SVC. Additionally, tenfold cross-validation was used with stratified sampling to tune the hyperparameters. The hyperparameters of each model and their ranges are shown in Table 5. This manuscript implements a tenfold cross-validation approach for tuning all hyperparameters. The training dataset is divided into 10 subsets, each following the same class distribution as the original class, called stratification which helps prevent biases in the learning process. 9 subsets are used to train the model, and the last subset is used as validation. This process is repeated till all the subsets have been used for validation once i.e. 10 times for each parameter combination. The accuracy is averaged out for these 10 combinations. This single iteration is repeated for all hyperparameter combinations. We used accuracy as an optimization metric for this process since we are trying to maximize it. The hyperparameters with the highest accuracy are then chosen and applied to the model which is then used on the training set and tested on the test set. The test set is unseen by the model until all the hyperparameters are tuned.

**Table 5:** An overview of the hyperparameters.

Model	Hyperparameter	Range
XGBoost	Learning rate	[0.1, 0.3]
	N estimators	[100, 300]
	Max depth	[3, 5, 7]
	Subsample	[0.6, 0.8, 1.0]
	Colsample bytree	[0.6, 0.8, 1.0]
KNN	N neighbors	[3, 5, 7, 9, 11]
	Weights	[‘uniform’, ‘distance’]
	Algorithm	[‘auto’, ‘ball_tree’, ‘kd_tree’, ‘brute’]
	p	[1, 2]
SVM	C	[0.1, 1, 10, 100]
	Kernel	[‘linear’, ‘poly’, ‘rbf’, ‘sigmoid’]
	gamma	[‘scale’, ‘auto’]
	degree	[2, 3, 4, 5]
Random forest	N estimators	[100, 300]
	Max depth	[None, 10, 20]
	Min samples split	[2, 5]
	Min samples leaf	[1, 2]

##### XGBoost

XGBoost is a gradient-boosting ML algorithm used for classification in this study. It builds an ensemble learning model by combining weaker learners and employs tree pruning to reduce complexity and prevent overfitting. XGBoost was chosen as the base learner due to its speed, performance, and versatility^36^.

##### Random Forest

Random forest constructs diverse decision trees by randomly selecting features at each split, reducing the correlation between trees and improving the model’s robustness. Due to its versatility, ability to handle complex data, and resistance to overfitting, random forest is a key classification model used in this study^37^.

At their core, both XGBoost and RF are based on decision trees. Decision trees divide the training examples into smaller trees until a pattern is found. There is a change in the entropy when these trees are divided into smaller groups. This change in the entropy is known as the information gain and is given by

3 $$\begin{aligned} Entropy(S) = \sum _{t=1}^{c} - (P_{t}\log _{2} P_{t}) \end{aligned}$$

4 $$Information\;Gain(S,A) = Entropy(S){\mkern 1mu} - \sum\limits_{{v \in Values(A)}} {\frac{{|S_{v} |}}{{|S|}}} \cdot Entropy(S_{v} )$$

##### KNN

KNN is a simple ML algorithm for classification and regression. It memorizes the entire training data and predicts the label for new data based on the majority vote of its K nearest neighbours. To determine these neighbours, KNN employees use distance metrics like Euclidean and Manhattan distance^38^.

##### SVM

SVM is a power ML algorithm used for both regression and classification. It aims to find the best hyperplane (separation line) by maximizing the distance to the closest support vectors (data points). SVM uses kernel operations like radial basis functions to create new dimensions and find the optimal hyperplane^39^.

### Experiment 2: Proposed clustering-based approach

The second experiment was divided into three phases based on the task performed. These were the feature generation, model implementation, and eXplainable AI (XAI) phases. The first phase implemented principles of unsupervised machine learning and generated an additional feature. K-centroid clustering techniques were used at multiple stages in the model. The pre-clustered data was used in the third phase to train the same ML models from the section “Implementing ML models”.

Further, the hold-out data underwent the same feature extraction process to assess the model’s effectiveness. Finally, XAI tools were employed to comprehend the impact of individual features on the predictions of the best-performing model. SHapley values were used to explain the model.

**Fig. 2:**
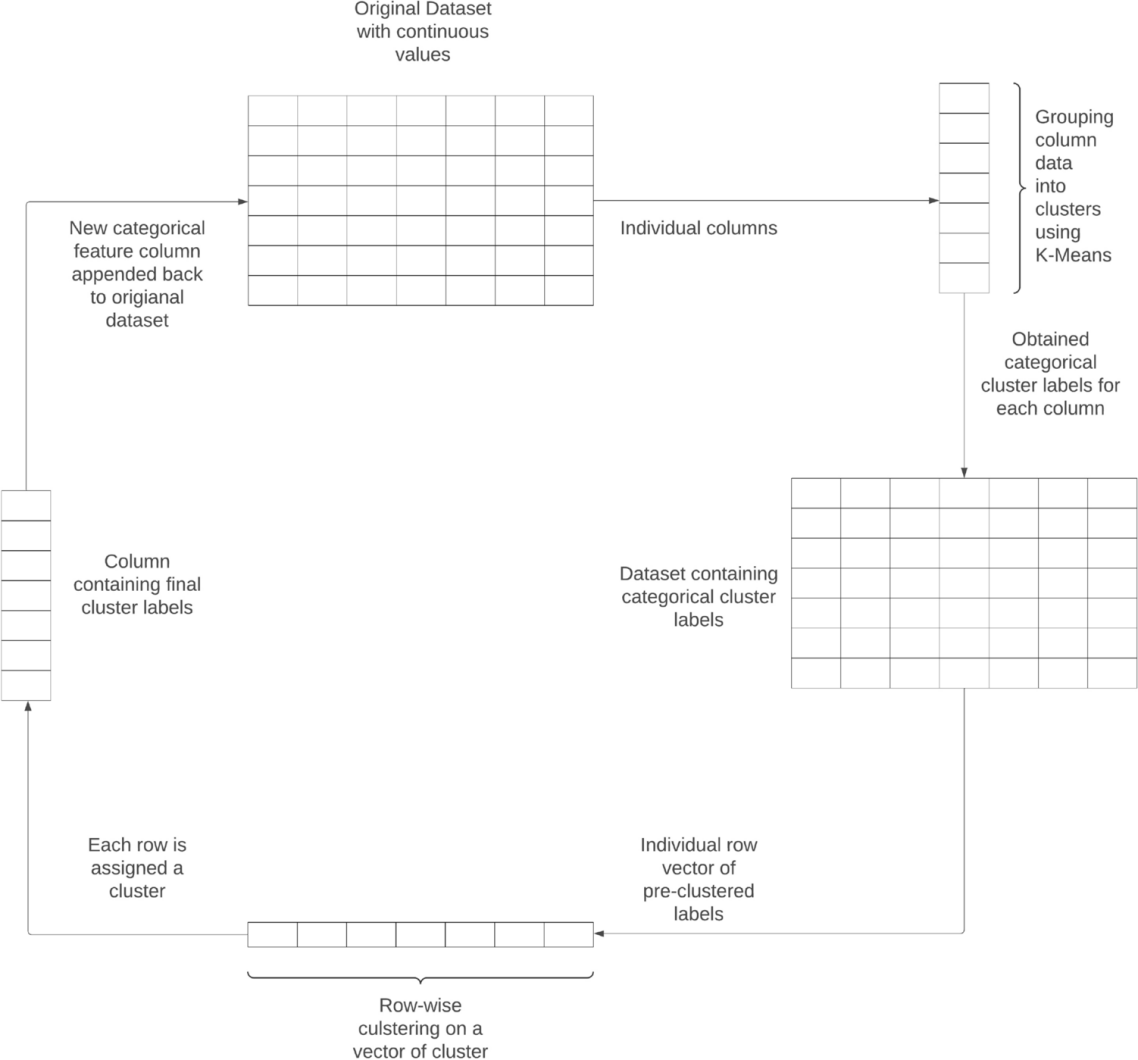
Diagrammatically elaborating on the feature generation process.

Notably, the test-train split was performed before beginning the clustering process. The test data was held out until training the learning model on the training set. The test set undergoes an identical process as the training dataset. We perform splitting before further processing the data to prevent data leakage from the test set to the training set. This preventive measure is taken to avoid the model being influenced by future data from the test set. Doing this provides a higher likelihood of generalizability and lowers the chances of an overly optimistic result. All the clustering steps were repeated for the test set with the same parameters as the training set. Figure 2 shows this entire process diagrammatically.

The sections “Clustering for production of categorical bins” and “Clustering for feature extraction” further elaborate on the phases.

#### Clustering for production of categorical bins

Clustering is a machine learning technique that segregates subsets of a dataset into groups based on commonalities and patterns in their attributes. It is used to gain essential insights from datasets that might go unnoticed. This unsupervised technique forms the crux of this research. It is used in two stages, first for binning individual features (columns) and second for predicting clusters for the entire binned dataset on individual patient entries (rows). This multidimensional clustering (row-wise and column-wise) on the dataset helps reduce complicated (noisy) data into a more interpretable version by assigning specific clusters.

Binning is the process of converting continuous numeric data into its categorical counterparts. This process involves splitting numerical values into appropriate ranges and providing a categorical label for each range. Categorizing data into bins increases the interpretability and reduces noise or non-linearity. This is essential as it sets the base for the following step. We use k-means clustering to predict the cluster bins for each parameter. The underlying data are reports from laboratory tests, thus well-defined and separated. K-means clustering performs well on such spherical or hyperspherical data and is thus used in this step. Also, it displays the best performance when compared to other k-centroid approaches.

**Fig. 3:**
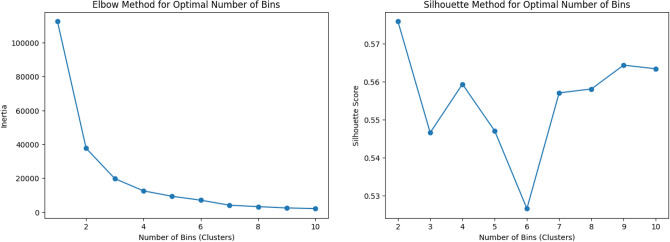
The plot on the left shows the elbow plot to find the optimal number of clusters. The point where the elbow is formed is the optimal number of clusters. The plot on the right shows the plot of silhouette scores to find an optimal number of clusters. A high amount of silhouette score is better.

Clustering is only effective if an optimal number of clusters are formed. In this study, we make an optimal number of bins or clusters by analysing the elbow curve and silhouette scores for up to ten clusters. These are graphical methods for evaluating cluster optimality by using the within-cluster sum of squares and the cluster cohesion or separation, respectively. Figure 3 depicts the elbow plot and silhouette scores for the variable “Age”. Once the optimal number of clusters for a given feature is decided, the feature is split up at the acquired optimal indices. This process is repeated for each feature in the dataset. The resulting dataset contains only categorical values.

#### Clustering for feature extraction

**Fig. 4:**
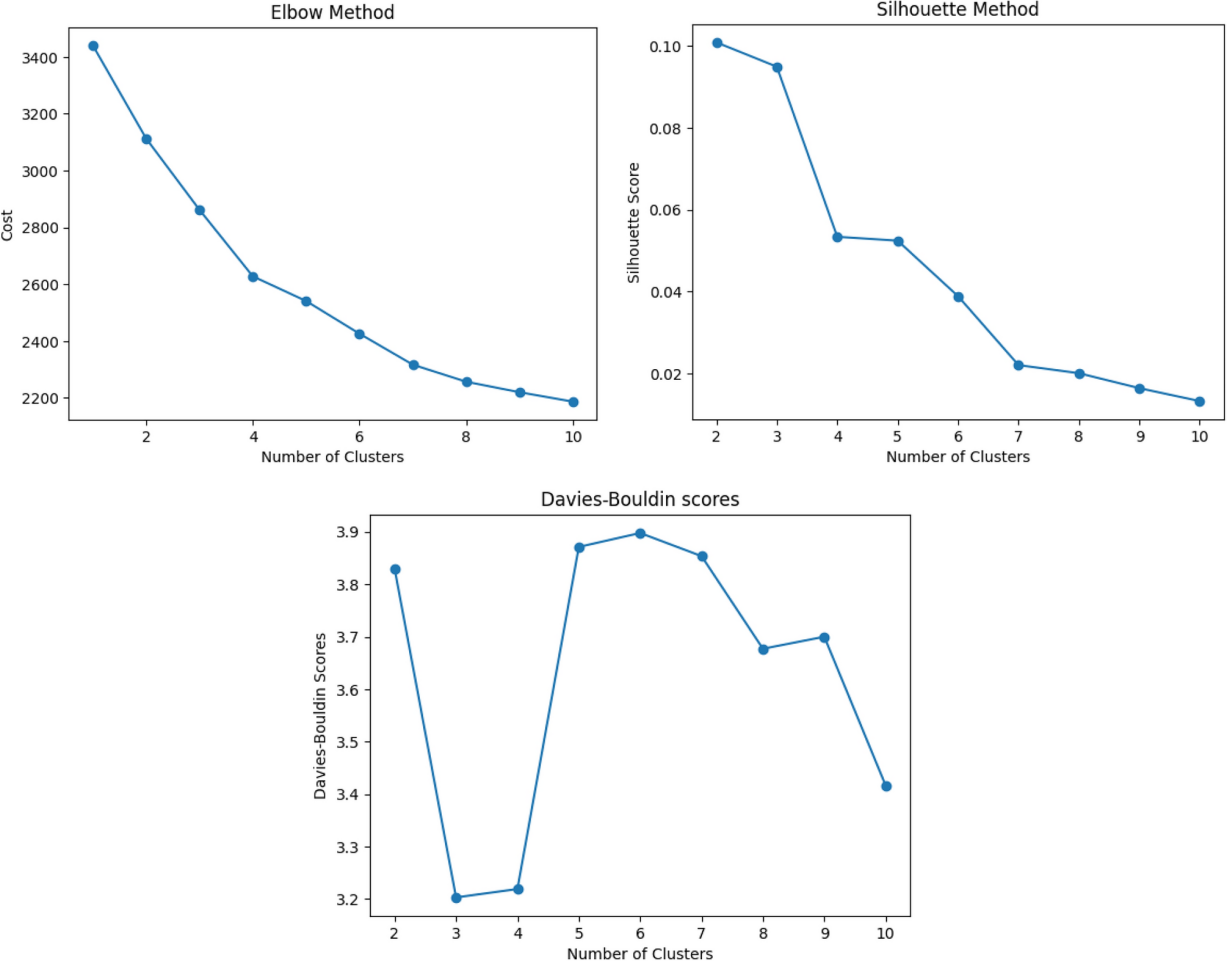
Silhouette score: higher is better, DB score: lower is better.

Since all the features are categorical, k-modes is the only feasible clustering approach. It uses the most frequent occurrence of a particular label to determine the clusters. The optimal number of k-mode clusters is also found using the elbow and silhouette methods. Additionally, we use the Davies-Bouldin (DB) scores to help us improve the estimation of the optimal clusters. The DB scores represent the mean similarity measure between each cluster and its most similar cluster, with the similarity measure determined by comparing distances within clusters to those between clusters and then averaging these ratios across all clusters. A lower score is better as it depicts clusters farther apart and evenly spread out. The three graphical evaluation methods are presented in Fig. 4.

The Cao method is used for cluster initialization. It was introduced by Cao et al.^40^ in 2009 and is one of the most popular cluster initialization methods. This method selects the initial cluster centres based on the distances between the objects and the density of each object. This study also found this initialisation method to give better results than its predecessors. This second clustering step introduces a new parameter, “kmodes_predicted”, using the intermediary binned data obtained in the previous step. This new parameter further increases the interpretability of the dataset, aiding the model performance. The new parameter is then appended to the original dataset, which contains continuous values, thus concluding the clustering phase. This new feature includes the collective underlying information from the categorical data, vital to improving model accuracy.

#### Explainable AI

XAI or eXplainable AI refers to a set of methods for interpreting the inner mechanisms of an AI model. Understanding the reason behind the prediction is usually difficult and creates a trust deficit. This section attempts to demystify the black-box operations proposed by the method proposed in this study.

##### SHapley additive exPlanations

This study utilized SHAP values to understand each feature’s contribution to the final prediction. SHAP utilizes concepts from cooperative game theory and SHapley values. For a given coalition, SHapley values determine the average contribution of a player (or feature) to the result (or prediction). The intuition behind these values is the analysis of the difference in the performance of a model with and without a particular feature or subsets of features. For a feature x, the contribution of x for each subgroup that includes x is calculated and averaged over all those contributions. This gives a marginal contribution of a feature to the feature set.

5 $$\phi _{i} (\nu ) = \frac{1}{{{\text{no}}{\text{.}}\;{\text{of}}\;{\text{players}}}}\sum\limits_{{{\text{coalitions}}\;{\text{including }}i}} {\frac{{i'{\text{s}}\;{\text{marginal }}\;{\text{contribution}}\;{\text{ to}}\;{\text{ coalition}}}}{{{\text{no}}{\text{.}}\;{\text{ of}}\;{\text{ coalitions}}\;{\text{ excluding}}\;i\;{\text{ of }}\;{\text{this}}\;{\text{ size}}}}}$$

##### Observations

**Fig. 5:**
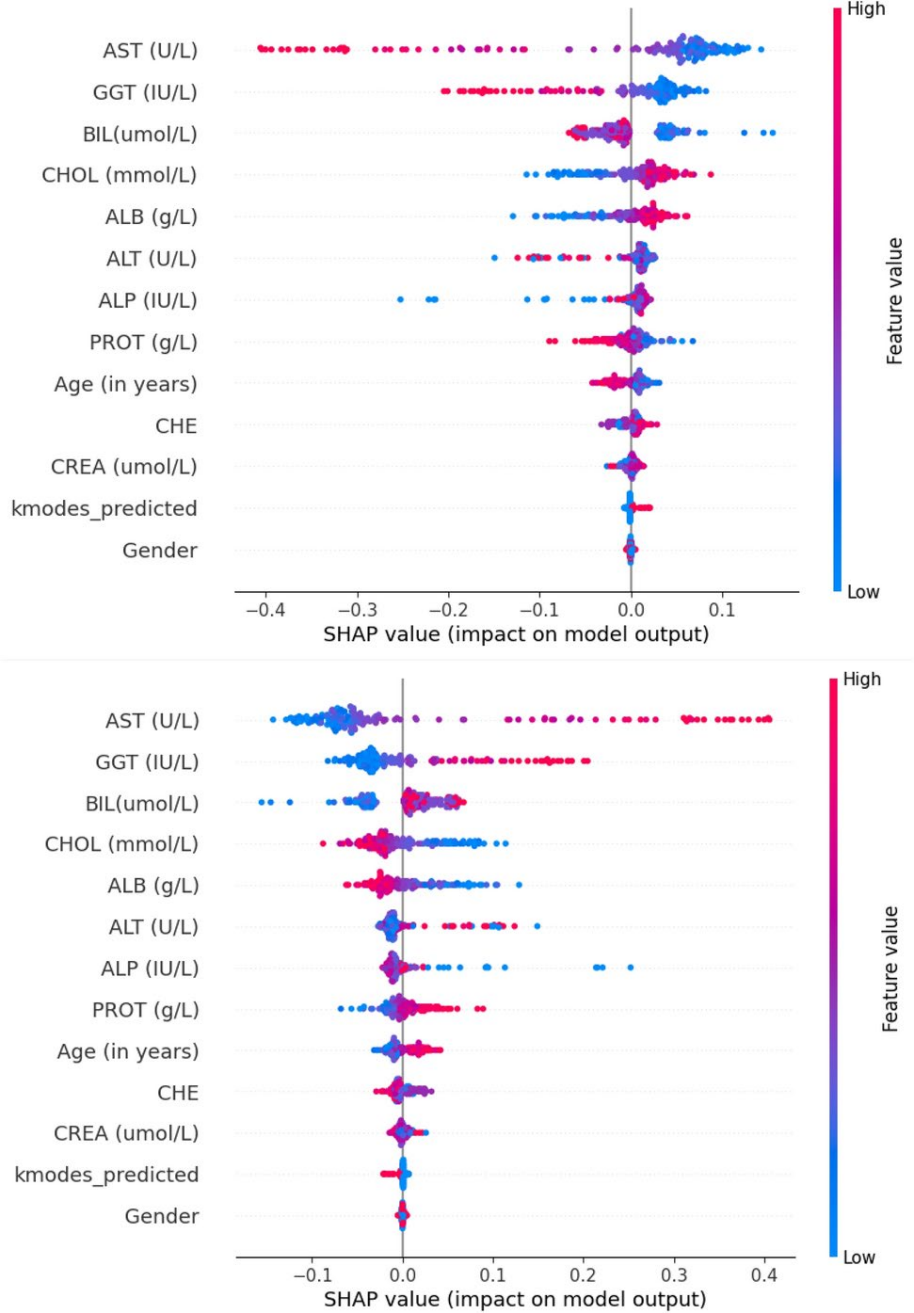
Beeswarm plot of class 1 (top) and class 0 (bottom) for the first instance in the test set. Since this is a binary classification, they are the inverse of each other.

Using SHAP, the beeswarm plot is obtained using the best-performing model. This plot is generated for class 0, which corresponds with the blood test reporting positive for the presence of HCV, and vice versa represents class 1. This plot provides a global understanding of the data. It visualizes the distribution of SHAP values (impact on the prediction) for a feature’s high or low values over all instances in the testing data. Each point represents a feature, and its position on the plot corresponds to its SHAP value. The plot is shown in Fig. 5, and the observations drawn are further explained in the upcoming paragraphs.

The observations drawn from the plot are as follows. We notice that Aspartate transaminase (AST), Gamma-glutamyltransferase (GGT), Bilirubin(Bil), Cholesterol (CHOL), and Albumin (ALB) are the five major contributors in the predictive output of the model. Further, it is noted that increased levels of AST, GGT, and Bil positively impact prediction for class 0, whereas decreased levels positively impact prediction for class 1. This implies that the value of AST, GGT, or Bil directly contributes to the likeliness of the patient suffering from HCV. Previously published studies also support this^41–43^.

Furthermore, the converse is observed for the values of CHOL and ALB. As the values of these features increase, a negative impact is generated on the output of class 0, while a positive impact is seen towards the prediction of class 1. This suggests an inverse relation between these features and the model output. This has also been observed in previous biological studies regarding these features^44,45^.

In this experiment, we chose two output models from the section “Experiment 2: Proposed clustering-based approach” to build a stacked meta-model. The models were selected based on their performance in the previous section. A third model (RF) was the final estimator, which combined the base models. The following section elaborates further on the proposed model.

#### Stacked meta-model

A meta-model is a higher-level model that combines the outputs of multiple machine learning models, called base models, into a single framework. A meta-learner generates the final predictions by training on the outputs of base ML models. This approach can be particularly practical when the base models exhibit complementary behaviors. By leveraging the learning capabilities of the meta-model, we further enhance the performance of the base models by reducing their biases. This study proposes a meta-model for the detection of hepatitis C. This model uses a stacking ensemble method. The base models used are the XGBoost and Random forest models obtained from the section “Experiment 2: Proposed clustering-based approach”. The meta-model in a stacking classifier gives the final output using the base models. In the proposed model, we use a random forest model as the meta-model. This stacking classifier was optimized sequentially. The random forest model is optimized while the XGBoost model is not. This approach is followed as it yields the highest accuracy from our tests.

### Ethical approval

This article contains no studies with human participants or animals performed by authors.

## Performance evaluation metrics

The performance metrics used to assess all the models in this research are: accuracy, precision, recall, and F1 scores, and the area under the receiver operating characteristics (ROC) curve^46^. These metrics are calculated using true positive (TP), true negative (TN), false positive (FP), and false negative (FN) values. A “confusion matrix” is created to represent these metrics visually.

### Accuracy

This indicates the blood samples that were correctly predicted. The accuracy is obtained from Eq. (6).

6 $$\begin{aligned} \text {Acurracy} = \frac{\text {TP + TN}}{\text {TP + FP + TN + FN}} \end{aligned}$$

where TP represents the count of correctly classified positive results, TN represents the count of correctly classified negative samples, FP represents the count of negative samples classified as positive, and FN represents the count of positive samples classified as negative.

### Precision

Precision is defined as the possibility of accurately classifying a positive blood sample. Precision is obtained from Eq. (7).

7 $$\begin{aligned} \text {Precision} = \frac{\text {TP}}{\text {TP + FP}} \end{aligned}$$

### Recall

Recall demonstrates the sensitivity of a model towards identifying the positive blood sample. Recall is obtained from Eq. (8)

8 $$\begin{aligned} \text {Recall} = \frac{\text {TP}}{\text {TP + FN}} \end{aligned}$$

### F1-score

F1-score integrates the recall and precision into a single metric using their harmonic mean to understand the overall model performance better. F1-scores are obtained from Eq. (9)

9 $$\begin{aligned} \text {F1-score} = 2 \times \frac{Eq.\,(7) \times Eq. (8)}{Eq.\,(7) + Eq.\,(8)} \end{aligned}$$

### Area under the ROC curve

By plotting sensitivity against 1-specificity for a model across various thresholds, the ROC curve effectively distinguishes between the ’signal’ and the ’noise’. The area under this curve measures the capacity of the given model to discriminate between positive and negative blood samples, providing a summary of its performance.

## Results and discussions

**Fig. 6:**
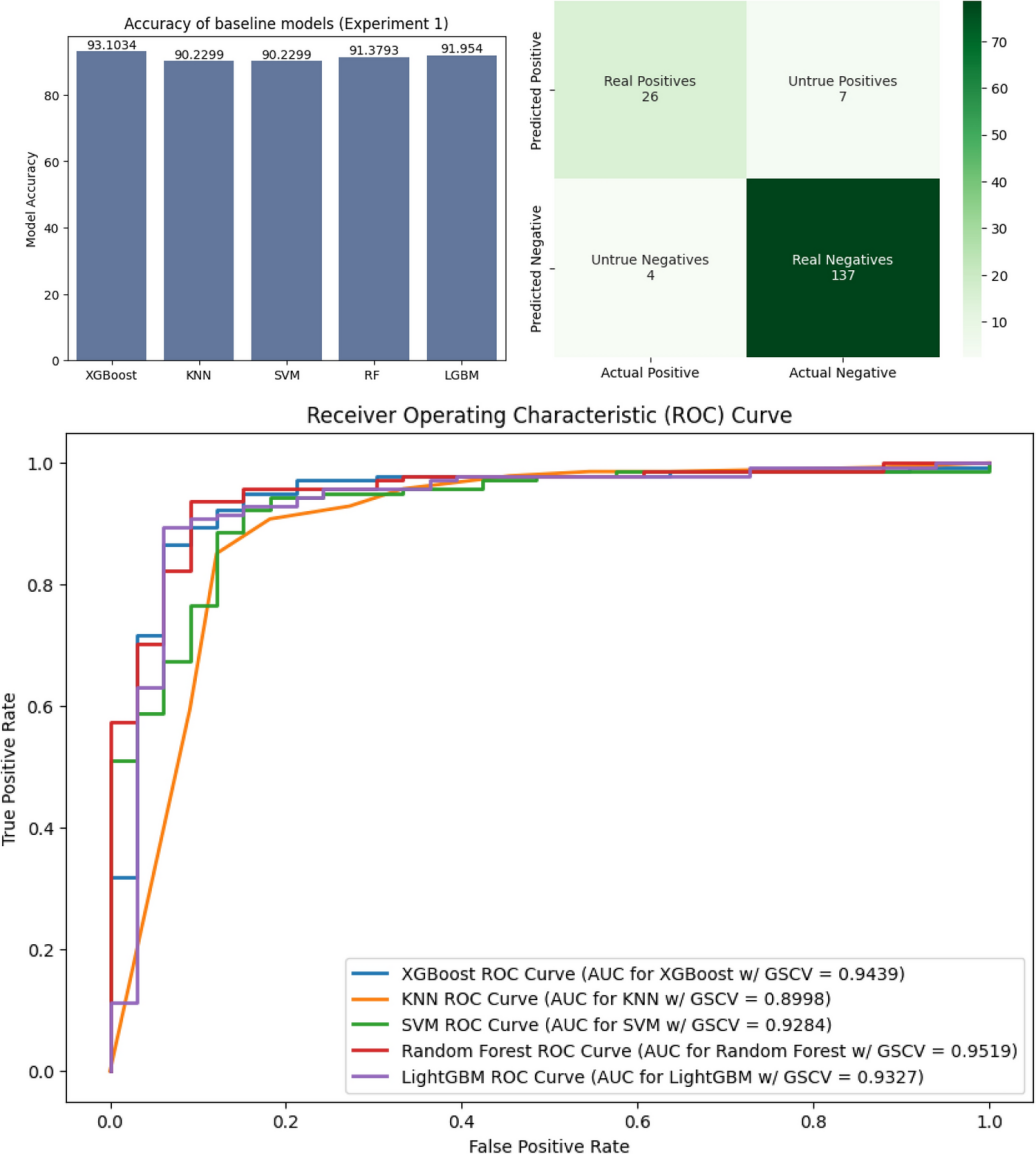
Diagram depicting the accuracy and ROC curve of the individual models along with the confusion matrix of the best-performing model for the baseline models.

**Fig. 7:**
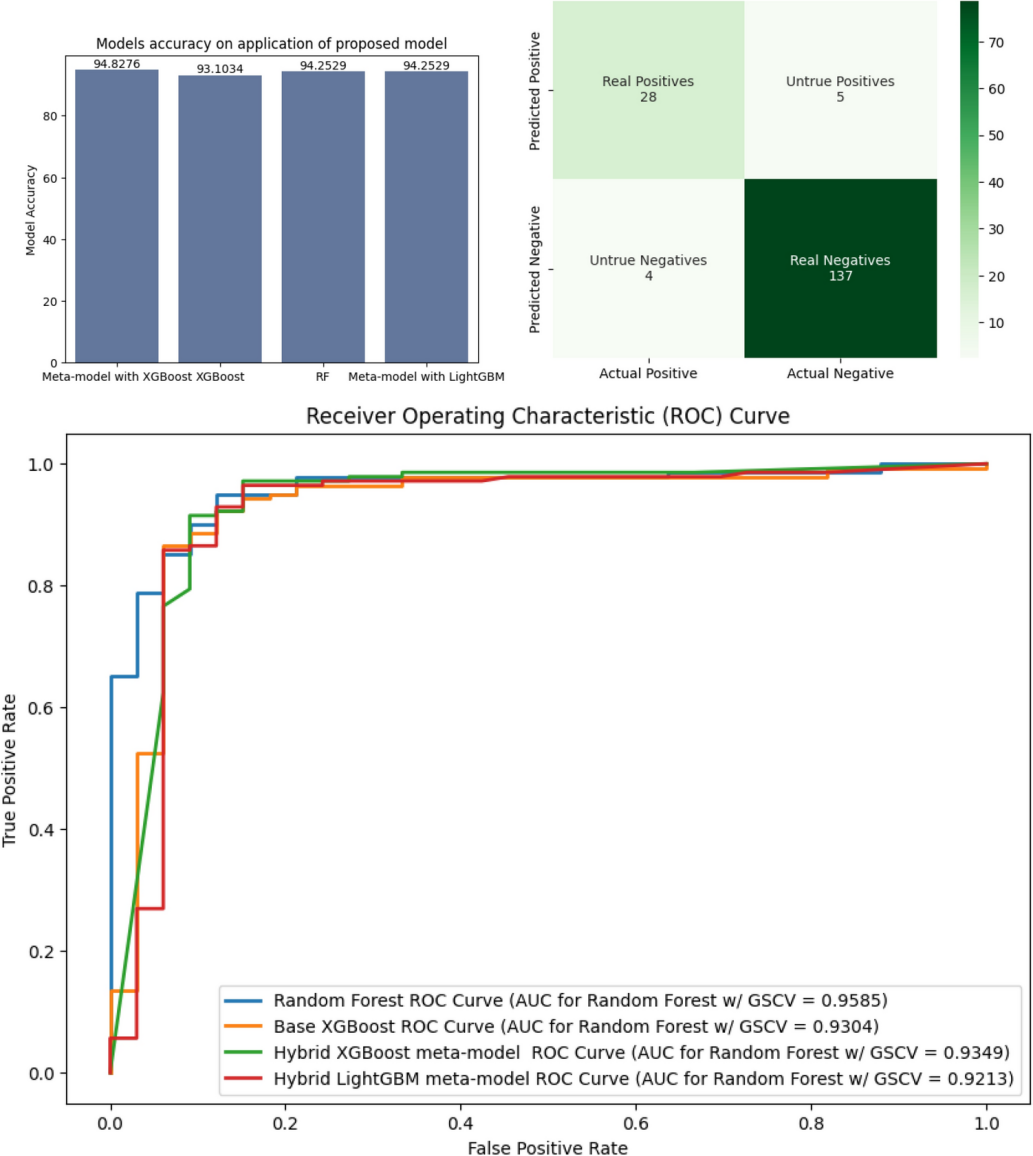
Diagram depicting the accuracy and ROC curve of the individual models along with the confusion matrix of the proposed meta-model.

The accuracy, area under the ROC curve, and confusion matrix for the first experiment are presented in Fig. 6. The same for the second experiment is displayed in Fig. 7 respectively.

The proposed meta-model demonstrated a 94.8276% accuracy in predicting the correct outcome, surpassing the best-performing baseline score of 93.6782% achieved by random forest without pre-clustering. The leading baseline model (RF) achieved a precision of 87%, recall of 79%, and f1-score of 83% for class 0 (positive HCV), while for class 1 (negative HCV), precision was 95%, recall was 97%, and f1-score was 96%. The ROC curve exhibited an area of 0.9609.

After the application of our proposed pre-clustering technique, the random forest achieved precision, recall, and an F1-score of 90%, 79%, and 84%, respectively for class 0. For class 1, precision, recall, and f1-scores were 95%, 98%, and 97%, respectively. The area under the curve achieved by the same model is 0.9585. From the above experiments, the performance of the random forest model has improved significantly.

Making a stacked meta-model using the previously obtained XGBoost and RF models, we obtained an accuracy, of 94.8275%, which is much better than the results of the previous two experiments. The class 0 precision, recall, and f1-scores were 88%, 85%, and 86%, respectively. The same values for class 1 were 96%, 97%, and 97%, respectively. Tables 6 and 7 have depicted these values in tabular form.

**Table 6:** Baseline results.

Model	Accuracy	Class	Precision	Recall	F1-score
XGBoost	93.1034%	0	84%	**79%**	81%
		1	**95%**	96%	**96%**
KNN	90.2299%	0	74%	76%	75%
		1	94%	94%	94%
SVC	90.2299%	0	77%	70%	73%
		1	93%	95%	94%
RF	**93.6782%**	0	**87%**	**79%**	**83%**
		1	**95%**	**97%**	**96%**
LGBM	92.5287%	0	83%	76%	79%
		1	94%	96%	95%

Significant values are in bold.

**Table 7:** Proposed method results.

Model	Accuracy	Class	Precision	Recall	F1-score
XGBoost	93.1034%	0	84%	79%	81%
		1	95%	96%	96%
KNN	90.8046%	0	82%	70%	75%
		1	93%	96%	95%
SVC	90.8046%	0	77%	73%	75%
		1	94%	95%	94%
RF	94.2529%	0	**90%**	79%	84%
		1	95%	**98%**	97%
LGBM	93.1034%	0	82%	82%	82%
		1	**96%**	96%	96%
Meta-model	**94.8276%**	0	88%	**85%**	**86%**
		1	**96%**	97%	**97%**

Significant values are in bold.

### Ablation study

We performed two ablation studies in this manuscript to analyze the impact of individual modules and stages in our methodology. The following sub-sections elaborate on these studies along with the results obtained which support the efficacy of our proposed approaches.

#### Proposed model efficacy study

This study conducted three ablation studies to observe the influence and contribution of the various modules on the overall performance of the proposed method.

First, we observe a decline in performance when removing only the meta-model classifier (denoted “w/o meta”). This shows that including the meta-model is significant in our stacked approach.

Second, a drop in the model performance is noted when hyperparameter tuning is not implemented (denoted “w/o HPMT”). This validates the significance of using stratified sampling and cross-validation sets to find the hyperparameters that perform the best.

Finally, when we remove the entire proposed approach (denoted “w/o prop.”), The model performance was significantly reduced, including pre-clustering and using a meta-model, which is directly attributed to our proposed approach.

The results of this study are depicted in Table 8.

**Table 8:** Results of proposed model efficacy study.

Models	Accuracy	Precision	Recall	F1
Proposed model	**94.82%**	**96%**	97%	**97%**
w/o meta	94.25%	95%	**98%**	97%
w/o HPMT	93.10%	95%	96%	96%
w/o prop.	93.67%	95%	97%	96%

Significant values are in bold.

#### Preprocessing impact study

Another ablation study performed was based on preprocessing. The results of excluding or changing individual preprocessing steps are given in Table 9. We note that using a mean imputation instead of a median yields a loss of 1.2% accuracy, excluding the outlier treatment process costs us 1.7% in accuracy, while without scaling there is seemingly no loss of accuracy in models that have a built-in scaling function. However, the absence of scaling in the models that lack this function is around 10% in accuracy as well as an exponentially higher run time of over 6 times more than the other models.

**Table 9:** Results of preprocessing impact study.

	Imputation	Outlier	Scaling	Best result
Original	Median	Yes	Yes	94.8%
Test 1	Mean	Yes	Yes	93.6%
Test 2	Median	No	Yes	93.1%
Test 3	Median	Yes	No	94.8%

## Threats and limitations

The proposed model has two threats and limitations. It lacks validation from real-world data, making it challenging to determine its practical performance. Moreover, preprocessing significantly influences the quality of the training model. This model is heavily dependent and sensitive to preprocessing, so improper implementation of preprocessing could threaten the proposed model.

## Conclusion and future works

The lack of a vaccine and the severity of advanced stages make HCV a worldwide issue. The symptoms of the disease are often subtle, and by the time one notices them, the condition is almost chronic. The existing medical tests are time-consuming and expensive, rendering them out of reach for most individuals. The disease can be treated orally if detected within 8–12 weeks of diagnosis. The early detection of HCV is crucial for prognosis and to provide timely care. ML models trained on data acquired from a standard biochemistry test provide rapid and inexpensive solutions. Various ML-based approaches are utilized to accurately predict HCV infection to reduce the burden on the medical infrastructure. This study proposed an unsupervised pre-clustering approach to a hybrid dataset to fill in the gaps left by previous research. The clustering approach was first applied to bin individual features and later the entire binned dataset for feature extraction.

Additionally, a meta-model was utilized to stack the base ML models to maximize the convergence and model’s accuracy. Two experiments are performed to assess the proposed model’s performance systematically. Analysing the results of our experiments, we observed that the clustering methods and proposed stacking meta-model displayed the highest accuracy and addressed the issues of generalizability.

A limitation of the model proposed in the study is the absence of real-world validation to assess its efficacy. Testing the model on real-world datasets would offer future research and improvement opportunities. Another area for future research could be exploring the effect of outliers on the predictions. Future works can explore the prediction of stages of HCV infection.

## Data Availability

The datasets generated during and/or analyzed during the current study are available from the corresponding author upon reasonable request.
